# Improvement of an antibody-enzyme coupling yield by enzyme surface supercharging

**DOI:** 10.1186/s12896-014-0088-6

**Published:** 2014-10-18

**Authors:** Agneta A Prasse, Thomas Zauner, Karin Büttner, Ralf Hoffmann, Thole Zuchner

**Affiliations:** Institute of Bioanalytical Chemistry, Faculty of Chemistry and Mineralogy and Centre of Biotechnology and Biomedicine, Universität Leipzig, Deutscher Platz 5, 04103 Leipzig, Germany; Current address: Octapharma Biopharmaceuticals GmbH, Im Neuenheimer Feld 590, 69120 Heidelberg, Germany

**Keywords:** ELISA, Human enteropeptidase light chain, Protein-protein coupling, Surface supercharging

## Abstract

**Background:**

Protein cross-coupling reactions demand high yields, especially if the products are intended for bioanalytics, like enzyme-linked immunosorbent assays. Amongst other factors, the coupling yield depends on the concentration of the proteins being used for coupling. Protein supercharging of enzymes can increase the solubility dramatically, which could promote enzyme-antibody coupling reactions. A highly soluble, supercharged variant of the enzyme human enteropeptidase light chain was created by a site-directed mutagenesis of surface amino acids, used for the production of an antibody-enzyme conjugate and compared to the wild type enzyme.

**Results:**

Wild type and mutant enzyme could successfully be cross-coupled to an antibody to give antibody-enzyme conjugates suitable for ELISA. Their assay performances and the analysis of the enzyme activities in solution demonstrate that the supercharged version could be coupled to a higher extent, which resulted in better assay sensitivities. The generated conjugate, based on the supercharged enzyme, was feasible as a reporter molecule in a sandwich ELISA and allowed the detection of epidermal growth factor with a detection limit of 15.63 pg (25 pmol/L).

**Conclusion:**

The highly soluble, surface supercharged, human enteropeptidase light chain mutant provided better yields in coupling the enzyme to an antibody than the wild type. This is most likely related to the higher protein concentration during the coupling. The data suggest that supercharging can be applied favourably to other proteins which have to be covalently linked to other polymers or surfaces with high yields without losses in enzyme activity or specificity.

**Electronic supplementary material:**

The online version of this article (doi:10.1186/s12896-014-0088-6) contains supplementary material, which is available to authorized users.

## Background

The extraordinary feature of enzymes to catalyze reactions with a high selectivity is utilized in a broad field of applications, including the paper-, food- and pharmaceutical industry [1]. Besides this, enzymes are used as bioanalytical tools, such as in biosensors or as amplifying agents in immunoassays. One prominent application is the enzyme-linked immunosorbent assay (ELISA), where an antibody-enzyme conjugate is used for the detection and quantification of analytes. Due to its compatibility to complex samples, this approach became a method of high significance in routine analysis and research [2]. Most applications have in common, that the enzyme has to be immobilized. Whilst adsorption, entrapment and covalent-cross coupling procedures are feasible for continuous flow-through processes in industry [3], the latter is essential for the production of ELISA conjugates. The application in this type of immunoassay requires a synthesis protocol which ideally results in a conjugate with an enzyme:antibody ratio of at least 1:1, without causing side-products and, more importantly, remaining unconjugated antibody [4].

Different coupling strategies have been developed, especially for the popular reporter enzymes horseradish peroxidase, alkaline phosphatase and β-galactosidase. The coupling reaction for the first enzyme-antibody conjugate applied in ELISA was based on the homobifunctional linker glutaraldehyde [5,6]. Although the resulting enzyme-protein complex partially retains its enzymatic activity and immunological specificity, it suffers from the formation of inactive homo-conjugates and polymers [4]. Thus, heterobifunctional linker molecules have been developed, which can react to two different functional groups, such as amino groups, *cis*-hydroxyl-groups in carbohydrates and thiol groups. As the coupling efficiency is also dependent on the amount of coupling sites on the proteins [7], the modification should occur to a large extend. Additionally, high concentrations of proteins will result in higher yields of coupling product, since diffusion- and orientation-controlled ligation processes become more likely. Although the commonly used enzymes in ELISA allow a satisfactory performance for most applications, the adoption of other enzymes might be interesting in terms of substrate diversity and sensitivity.

In 2007, Lawrence *et al.* presented a method to decrease protein aggregation tendencies by specific mutations of residues on protein surfaces. This specific replacement of surface amino acids with either acidic or basic amino acids was termed *supercharging*, as the net charge of the mutants were more positive or negative [8]. Supercharged proteins have been engineered to overcome limitations of macromolecule delivery into mammalian cells [9] and to increase thermal stability of antibody fragments [10]. This strategy might also be useful to improve the yield of protein-protein coupling reactions, especially the linking of enzymes to antibodies for immunological applications.

We adapted supercharging to the light chain of the human enteropeptidase in an earlier study and the resulting mutant, hEPl scC112S (N6D, G21D, G22D, C112S, N141D, K209E), showed a superior solubility and heat stability [11]. Here, we investigated the impact of enzyme supercharging on chemical crosslinking as exemplified for an antibody-enzyme conjugate. Therefore, hEPl scC112S and its wild type variant (hEPl wt) were conjugated to a secondary antibody and compared with respect to their assay performance.

## Methods

### Reagents

Enzymes (hEPl wt and hEPl scC112S) were expressed, refolded and purified in-house [11]. Polyclonal sheep-anti-mouse and donkey-anti-rabbit antibodies were from Dianova (Hamburg, Germany), monoclonal mouse-anti-EGF, EGF protein and polyclonal rabbit-anti-EGF were from Abcam (Cambridge, UK). Citric acid and Tween®20 were from Serva Electrophoresis (Heidelberg, Germany), Tris(hydroxymethyl)-aminomethan was from Applichem (Darmstadt, Germany). C_6_-Succinimidyl-4-formylbenzoate and C_6_-Succinimidyl-4-hydrazinonicotinate acetone hydrazone were from Acris Antibodies (Herford, Germany). Sodium chloride, hydrochlorid acid (conc.), Na_2_HPO_4_x12H_2_O, KH_2_PO_4_, GD_4_K-na and DMSO were purchased from Sigma-Aldrich (Taufkirchen, Germany) in the highest purities available. Roti®-Block was ordered from Roth (Karlsruhe, Germany). Bovine serum albumin standard was from Thermo Fisher Scientific (Rockford, USA). Deionized water was obtained from a Purelab Ultra water purification system (ELGA LabWater, Celle, Germany; 18.2 MΩ*cm).

### Antibody-enzyme coupling

A 1.5-fold molar excess of enzyme (hEPl wt: 90 μg/mL or hEPl scC112S: 440 μg/mL) was coupled to a sheep-anti-mouse antibody (2.4 g/L) after dialysing the proteins against modification buffer (0.1 mol/L phosphate buffer, 0.15 mol/L NaCl, pH 8.0) separately for 16 hours at 4°C using a 12 kDa cut-off dialysis tube (Sigma-Aldrich). Afterwards, the antibody was mixed with 40 equivalents of C_6_-succinimidyl-4-formylbenzoate (9 g/L in DMF), while enzymes have been modified with 40 equivalents of C_6_-succinimidyl-4-hydrazinonicotinate acetone hydrazone (5.2 g/L in DMF). The reaction mixtures were incubated at room temperature for 3 hours and excess linker molecules were removed by dialysis against conjugation buffer (0.1 mol/L citric acid, 0.15 mol/L NaCl, pH 6.0) at 4°C. Antibody and enzyme solution were mixed and incubated at 4°C. After 20 hours, protein concentrations were determined using a Bradford assay according to standard protocols with bovine serum albumin as standard [12]. The solutions were used without further treatment.

The ELISA-conjugate was obtained by increasing the concentration of hEPl scC112S from 0.242 g/L to 3.6 g/L by lyophilisation from 5 mmol/L Tris-buffer (pH 8.0) and subsequent resolubilization of the enzyme in water. Coupling was performed as described above, using a donkey-anti-rabbit antibody (1.2 g/L) and a 10-fold molar excess of enzyme.

### Enzyme assay

Kinetic characterization of the enzymes was performed in a 0.1 mol/L Tris–HCl buffer (pH 8.0, 10% DMSO) at room temperature using black 384-well plates (non-binding, Greiner Bio-one, Frickenhausen, Germany). GD_4_K-na in concentrations varying from 3.7 to 947 μmol/L was cleaved by addition of hEPl wt and hEPl scC112S (final concentration 10 nmol/L). The linear increase of released 2-naphthylamine was recorded on a Paradigm fluorescence reader (Molecular Devices, Ismaning, Germany) using a fluorescence cartridge (λ_ex_ = 360/35 nm; λ_em_ = 465/35 nm; integration time 140 ms). All experiments were performed in triplicates.

### Enzyme-linked immunosorbent assay (ELISA)

ELISA experiments were performed in black 384-well microtitre plates (high-binding, Greiner Bio-one, Frickenhausen, Germany) with an assay volume of 100 μL. All experiments were performed in triplicates.

Initial testing of the conjugate yield derived from the coupling with 1.5 equivalents hEPl wt or hEPl scC112S was determined using an affinity test. 100 μL of an in-house produced monoclonal antibody (HPT-104 [13], 2.25 μg/mL) were incubated at 4°C for 16 hours. After a threefold washing step using 200 μL washing buffer (phosphate buffered saline, 10 mmol/L phosphate, 0.3 mol/L NaCl, pH 7.4, 0.05% (v/v) Tween®20), free binding sites were blocked with 120 μL blocking buffer (0.5% (w/v) casein in washing buffer). After a further washing step, conjugate solutions were added with a total protein concentration of 2 μg/mL and incubated. After removal of unbound components by washing, signals were generated by addition of 50 μmol/L GD_4_K-na in assay buffer (0.1 mol/L Tris–HCl, pH 8.0, 10% DMSO) and the fluorescence was monitored at 465 nm after excitation at 360 nm.

The microtitre plate for the EGF sandwich ELISA was coated with monoclonal mouse-anti-EGF antibody by incubating the solution (100 μL, 1 mg/L) at 4°C for 16 hours. After washing, blocking was performed with 120 μL Roti®-Block and the plate was washed again before the addition of a four-fold serial dilution of EGF (10 ng/mL to 39.1 pg/mL) in *E. coli* cell lysate (protein concentration 6 mg/L). After incubation for 1 hour and washing, 100 μL of a polyclonal rabbit-anti-EGF antibody (250 ng/mL) was added. Subsequently, the plate was washed and 100 μL of the diluted (1:1000) antibody-enzyme conjugate was incubated for one hour. After washing, 50 μmol/L GD_4_K-na (in 0.1 mol/L Tris–HCl, pH 8.0, 10% DMSO) was added for signal development. End-point measurement was performed 4 hours after incubation at 37°C with λ_ex_ = 360 nm and λ_em_ = 465 nm. All experiments were performed in triplicates.

## Results

**Figure 1:**
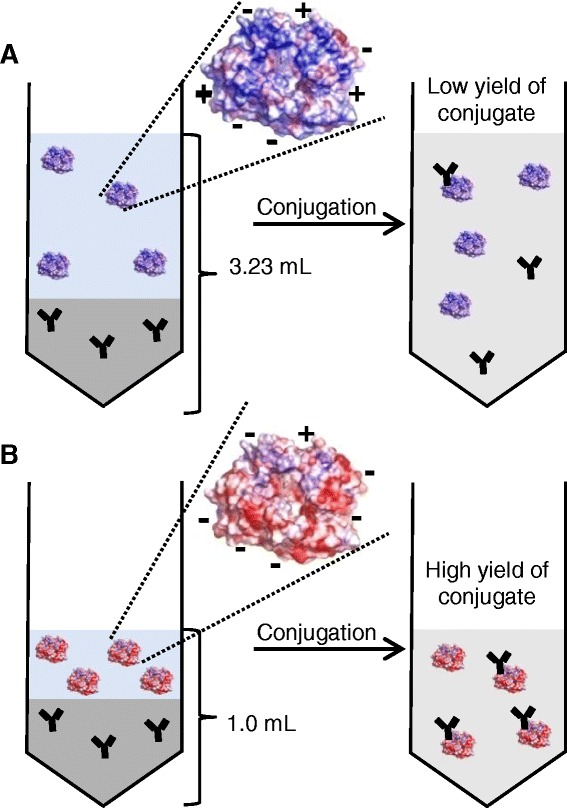
**Schematic principle of the improvement of a protein-protein coupling reaction by enzyme surface supercharging.** A high volume of wild type enzyme of the human enteropeptidase light chain is required due to its low solubility, resulting in a high total reaction volume and low yield of coupling product **(A)**. Highly soluble surface supercharged human enteropeptidase light chain permits an increase of the enzyme concentration prior to the coupling process **(B)**. Therefore the total reaction volume can be reduced to give a higher yield of desired product (antibody-enzyme conjugate).

Low reaction volumes are favoured for antibody-enzyme coupling reactions to obtain high coupling yields. Hence*,* proteins with high solubility are favoured for coupling*.* We therefore investigated, if the method of protein supercharging can produce mutants that are more suitable for coupling reactions compared to their wild type enzymes using human enteropeptidase light chain (hEPl) as a model enzyme (Figure 1). The light chain of the enteropeptidase holoenzyme, a 26 kDa enzyme-fragment, contains the catalytic triade and is therefore sufficient for most enzymatic applications. In an earlier study, a rational design of a mutant with a high surface charge (N6D, G21D, G22D, C112S, N141D, K209E) resulted in a more than 100-fold increase in enzyme solubility [11], which should affect the coupling reaction positively.

As a first step for the characterization of the enzyme, the kinetic parameters of the wild type and mutated enzyme were determined (Table 1 and Additional file 1). GD_4_K-na was chosen as a specific substrate, because its cleavage can be detected with high sensitivity (λ_ex_ = 360 nm, λ_em_ = 465 nm). The data indicate that the mutation of the enzyme resulted in a slightly decreased affinity towards the substrate, as the Michaelis constant *K*_m_ was increased from 0.11 mmol/L (hEPl wt) to 0.65 mmol/L for hEPl scC112S. The insertion of aspartic acid and glutamic acid in the mutant decreased the net charge of the enzyme by a difference of 6. This could result in electrostatic repulsion between enzyme surface and the tetra-aspartyl-motif of the substrate, and could be an explanation for the slightly lower substrate affinity of the supercharged enzyme hEPl scC112S. On the other hand, the mutant enzyme possesses an increased turnover number for GD_4_K-na, which confirms the data obtained in earlier studies for another substrate, Z-Lys-SBzl [11]. This means that at high concentrations of substrate (>1 mmol/L), the mutated variant hEPl scC112S would result in a faster signal increase upon digestion than the wild type enzyme.

**Table 1:** **Kinetic parameters for the digestion of GD**_**4**_ **K-na by hEPl wt and hEPl scC112S**

**Enteropeptidase**	***K***_**m**_ **[mmol/L]**	***k***_**cat**_ **[s** ^**−1**^ **]**	***k***_**cat**_ **/** ***K***_**m**_ **[(mmol/L)** ^**−1**^ **s** ^**−1**^ **]**	**Ref.**
hEPl wt	0.11 ± 0.01	81.9 ± 9.8	745	This study
hEPl scC112S	0.65 ± 0.09	169.3 ± 24.5	260	This study
Human recombinant light chain	0.16 ± 0.01	115 ± 5	719	[14]

**Figure 2:**
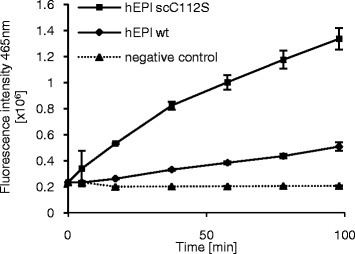
**Assay performances of hEPl wt and hEPl scC112S conjugate solutions.** Detection of an immobilized model antigen (HPT-104 antibody [13]) was performed using 2 μg/mL total protein of untreated conjugate solution. After incubation and washing, signal was generated by addition of 50 μmol/L GD_4_K-na and monitored for 100 minutes. Errors = SD, n = 3.

In order to analyze the coupling efficiencies of the two enzymes, both enzymes were coupled to a secondary antibody under the same coupling conditions and an ELISA experiment was carried out. Both enzyme conjugates led to signals that differed distinctly from the negative control. The signal increase for the supercharged conjugate (Ab-hEPl scC112S) was significantly higher than that for the wild type conjugate (Ab-hEPl wt) (Figure 2). While Ab-hEPl scC112S induced a signal increase of 15758 fluorescence intensity units per minute, application of the wild type conjugate resulted in a signal increase of only 2611 fluorescence intensity units per minute.

This indicates a higher yield of supercharged enzyme-antibody conjugate. Control experiments without antigen did not show any signal differing from the negative control (Additional file 2). As the used substrate concentration for the ELISA experiment in this study is much lower (50 μmol/L) than the concentration needed to reach v_max_ (1 mmol/L), the faster cleavage in the ELISA obtained here can only origin from a higher amount of enzyme-antibody conjugates.

This demonstrates that the application of enzyme supercharging generated a highly soluble mutant, which is more suitable for coupling processes than its wild type form. Unfortunately, we were not able to further confirm this result via native or denaturing SDS-PAGE or Western Blot. Both methods did not show clear bands that could be used for a quantification of the coupling product (data not shown).

**Figure 3:**
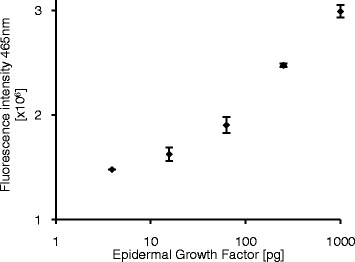
**Use of a hEPl scC112S-antibody conjugate for the detection of EGF.** Epidermal growth factor (in *E. coli* cell lysate as matrix) was detected using a sandwich ELISA and an in-house produced donkey-anti-rabbit/hEPl scC112S conjugate as secondary antibody. Signal was generated by addition of 50 μmol/L GD_4_K-na and an end-point measurement was performed after 4 hours of incubation at 37°C. The limit of detection (S/B >3xSD) was 15.63 pg EGF. Errors = SD, n = 3.

To further investigate possible bioanalytical applications of the synthesized conjugate, a coupling reaction with a 10-fold molar excess of enzyme (hEPl scC112S) was performed. The product was then utilized as a reporter molecule for the detection of epidermal growth factor (EGF). EGF is a low-molecular-weight polypeptide which affects many biochemical pathways by binding to its receptor and can be used as a cancer biomarker [15]. EGF was diluted in a complex mixture (*E. coli* cell lysate, 6 mg/L) and detected in a standard sandwich ELISA using two specific antibodies and the synthesized antibody conjugate (Ab-hEPl scC112S) was added for detection. The observed signals after addition of the peptide substrate GD_4_K-na were dependent on the amount of analyte present and permitted a detection of EGF down to 15.6 pg (Figure 3). This indicates that the hEPl scC112S mutant can be used as a reporter molecule in ELISA, while the wild type enzyme is not suitable for this application. This originates from the low solubility of the wild type which does not allow a high-yielding coupling protocol. In contrast to this, the supercharging surface modification of the enzyme results in an increased protein solubility as well as heat stability, which subsequently lead to higher yields of the desired antibody-enzyme conjugate.

## Discussion

Enzymes, like the serine protease enteropeptidase examined in this study, are widely used tools in biotechnology, industry and bioanalytics due to their catalytic function and their high specificity. However, many applications require the immobilization of these proteins on insoluble surfaces or on other biomolecules, such as other proteins. A very prominent example for enzyme-protein couplings are enzyme-antibody couplings and their subsequent use in enzyme immunoassays. As discussed earlier, various methods for enzyme-antibody couplings exist. However, the central problem of unsatisfactory protein solubility affects all of those coupling methods in a negative way.

One way to improve protein solubility is to optimize the buffer components of the coupling buffer by applying different ionic compounds or osmolytic stabilizers like sugars and polymers [16]. However, proteins are very sensitive to these conditions and slight modifications can cause protein precipitation, a loss in enzyme activity or antibody affinity. Other methods for the improvement of protein solubility exist, though they are not always suitable for the described problem. In 2009 Golovanov *et al*. showed, that protein solubility can be increased by a simple addition of charged amino acids. Addition of arginine and glutamic acid made several proteins accessible to NMR-studies by suppression of their aggregation tendencies [17]. Nevertheless, this method may not be suitable for protein-protein coupling reactions, since the additives possess the same reactive moieties as the proteins (e.g. amino groups). In case of protein modification with commonly used NHS-ester based linker molecules, linker reactivity would be quenched, as the reaction cannot occur specifically with the protein. This would decrease the protein modification degree and impact the overall coupling process negatively.

Also the introduction of a solubility-enhancement tag, as described in Lee *et al*. [18] and Zhou *et al*. [19] may have undesired effects, because it might alter the enzyme’s activity and selectivity. In principle, a mutation of surface amino acids of proteins can of course also lead to undesired side-effects. However, a careful design of suitable amino acids can leave the enzyme unaffected. This has been shown by us before for the mutation of surface amino acids of the human enteropeptidase light chain by site-directed mutagenesis [11].

Site-directed mutagenesis was found not only to be a tool for tailoring enzyme activity and stability, but also to improve immobilization reactions. Methods were reviewed in detail before and are for example based on the production of mutants that were capable of site-directed immobilization via specific tags [20,21]. Such methods include the incorporation of particular amino acid sequences for affinity fixation (anti-tag antibody to tagged enzyme, avidin to biotinylated protein) or the addition of a Lys-containing tags for the covalent linkage via transglutaminase cross-coupling.

Coupling procedures for the synthesis of antibody-enzyme conjugates require particularly high yields, since any unlabeled antibody fraction decreases the assay sensitivity, especially because it is difficult to purify the resulting coupling product from unmodified antibody. In addition to that, the preparation of highly pure and active enzymes is enormously laborious, which makes them an expensive material. Therefore high coupling yields are desired not only in terms of assay performance, but also with regard to material costs.

In this study, we were able to demonstrate that site-directed mutagenesis can be used to optimize coupling reactions due to an enhancement of the protein solubility. For that purpose, we tested the potential of a supercharged version of human enteropeptidase light chain with respect to its antibody-coupling yield and could show that the method of supercharging can evolve highly soluble proteins that are suitable for coupling reactions. As proven by us earlier, the site-directed surface modification of the human enteropeptidase light chain resulted in a more than 100-fold increased solubility which allowed the use of a highly concentrated enzyme solution without causing protein aggregation. This allowed the covalent linkage to an antibody with significantly higher yields compared to the wild type enzyme. Lee and coworkers demonstrated earlier that the resulting enteropeptidase-antibody conjugates are attractive components for heterogeneous immunoassays [22]. With the new coupling strategy, we showed here that the medium-scale synthesis of this kind of enzyme-antibody conjugates is possible, if a highly soluble supercharged version of the enzyme is used.

In summary, we could show that the supercharged human enteropeptidase light chain can be effectively used for the production of an antibody-enzyme conjugate. This procedure allows the use of the mutant enzyme as a reporter molecule in a sensitive ELISA system, while the wild type enzyme failed for this application. We furthermore suggest that this strategy can in principle be used for the coupling of any desired enzyme to other proteins. Examples of such enzymes which may be used for establishing novel ELISA systems may include subtilisin [23], acetylcholinesterase [24] and lipase [25].

## Conclusion

This study showed that surface supercharging of proteins is a suitable method for the improvement of protein-protein cross coupling reactions. Hence, the biotechnologically improved, highly soluble mutant hEPl scC112S could be utilized as a reporter molecule for a sensitive ELISA system. In contrast, the wild type enzyme could not be coupled to a satisfactory extend and is therefore not suitable for this application.

## Additional files

Additional file 1
**Characterization of enzyme kinetics for hEPl wt and hEPl scC112S.** Measurements were performed in 0.1 mol/L Tris-buffer (pH 8.0, 10% DMSO) with a GD_4_K-na substrate concentration range from 3.7 to 947 μmol/L. Enzymes were added to a final concentration of 10 nmol/L and fluorescence was recorded at 465 nm (λ_ex_ = 360 nm). *K*_m_ values were obtained from the fitted curves and were 0.11 mmol/L for hEPl wt and 0.65 mmol/L for hEPl scC112S. Errors = SD, n = 3. Additional file 2
**Negative controls in ELISA.** In the absence of antigen, no unspecific binding by both conjugate mixtures could be detected. Conjugate solutions with a total protein concentration of 2 μg/mL were used; signal development was carried out by addition of 50 μmol/L GD_4_K-na. Errors = SD, n = 3.

## Abbreviations

EGF: Epidermal growth factor
ELISA: Enzyme-linked immunosorbent assay
FI: Fluorescence intensity
hEPl: Human enteropeptidase light chain
*k*_cat_: Turnover number
*K*_m_: Michaelis constant
scC112S: Surface charged mutant
SD: Standard deviation
SDS-PAGE: Sodium dodecyl sulphate polyacrylamide gel electrophoresis
v_max_: Maximum rate
wt: Wild type
