# Supplementary figures and images for: Improvement of an antibody-enzyme coupling yield by enzyme surface supercharging
Source: BMC Biotechnol. 2014 Oct 18;14:88. doi: 10.1186/s12896-014-0088-6 (PMC4203919; doi:10.1186/s12896-014-0088-6)

$\Delta$  FI 465/min [ $\times 10^6$ ]

◆ hEPI wt

■ hEPI scC112S

0

250

500

750

1000

GD<sub>4</sub>K-na [ $\mu$ mol/L]

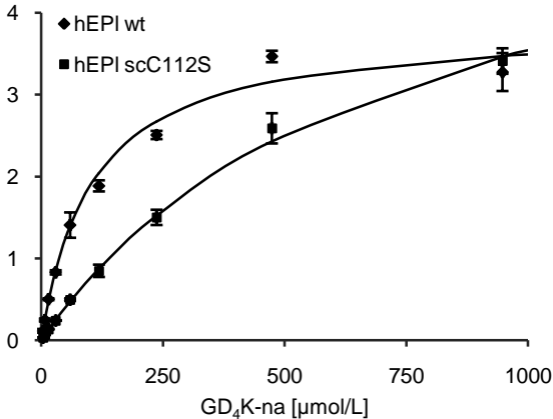

Supplement: Additional file 1 — Characterization of enzyme kinetics for hEPl wt and hEPl scC112S. Measurements were performed in 0.1 mol/L Tris-buffer (pH 8.0, 10% DMSO) with a GD4K-na substrate concentration range from 3.7 to 947 μmol/L. Enzymes were added to a final concentration of 10 nmol/L and fluorescence was recorded at 465 nm (λex = 360 nm). K m values were obtained from the fitted curves and were 0.11 mmol/L for hEPl wt and 0.65 mmol/L for hEPl scC112S. Errors = SD, n = 3. [file 12896_2014_88_MOESM1_ESM.pdf]

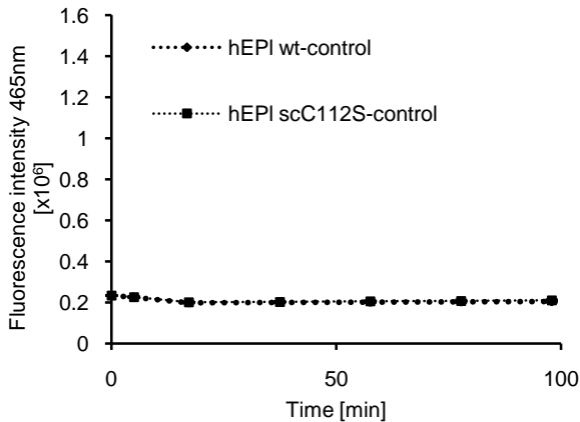

Supplement: Additional file 2 — Negative controls in ELISA. In the absence of antigen, no unspecific binding by both conjugate mixtures could be detected. Conjugate solutions with a total protein concentration of 2 μg/mL were used; signal development was carried out by addition of 50 μmol/L GD4K-na. Errors = SD, n = 3. [file 12896_2014_88_MOESM2_ESM.pdf]
